# Supplementary material for: Anisotropic mechanical properties of Dodecanophene nanosheets with pre-existing cracks by molecular dynamics simulation: Uncovering orientation- and temperature-induced variations
Source: PLoS One. 2026 Mar 6;21(3):e0339525. doi: 10.1371/journal.pone.0339525 (PMC12965698; doi:10.1371/journal.pone.0339525)
Supplement: Attachment — Submitted filename: Response Letter.docx [file pone.0339525.s002.docx]

PONE-D-25-51742

Thermal- and Defect-Induced Mechanical Behavior of Dodecanophene Nanosheets: Effects of Temperature and Crack Geometry from Molecular Dynamics Simulations

PLOS ONE

***Dear Editor***

We sincerely thank the reviewer for their thorough and constructive comments, which have significantly enhanced the quality and clarity of our manuscript. We have carefully addressed each comment with detailed responses, manuscript revisions, and additional supporting materials as outlined below.

***Additional Editor Comments:***

The manuscript must be revised based on the reviewers’ comments plus the following issues,

1) A separated file must be provided for the authors’ answers to the comments, one by one. Moreover, all changes must be yellow-colored highlighted sentences in the revised article. The track changes condition is not suggested.

***Answer:***

Dear Editor,

Thank you for your clear instructions regarding the revision process.

We confirm that we have prepared a separate response file containing point-by-point replies to each reviewer and editorial comment, as requested. In addition, all modifications made in the revised manuscript have been highlighted in yellow directly in the text to facilitate easy identification of changes. We have not used “Track Changes”; instead, only the final revised sentences/paragraphs that incorporate the requested corrections are highlighted.

Both the clean revised manuscript with yellow highlights and the standalone response letter are included in the resubmission package.

Thank you again for your guidance, and we appreciate the opportunity to improve our manuscript.

2) Using references suggested by the reviewers is not mandatory. If they are related, they could be used by the authors. If not, they could be ignored by the authors.

***Answer:***

3) It is not clear what the novelty is in the article title.

***Answer:***

Thank you for your valuable feedback on our manuscript. We agree that the original title did not sufficiently emphasize the novelty of our work, which lies in providing the first comprehensive molecular dynamics analysis of the anisotropic mechanical and fracture behavior of Dodecanophene—a recently proposed 2D carbon allotrope—under the combined influences of temperature, crack orientation, and defect size, revealing distinct directional dependencies, failure modes, and thermal sensitivities not previously explored for this material. To address this, we have modified the title accordingly.

4) All keywords must be found in the abstract or the title.

***Answer:***

The title and abstract were changed to include all keywords.

5) 10 references for the first paragraph of the introduction. It must be shortened.

***Answer:***

Thank you for your valuable feedback regarding the opening paragraph of the Introduction. In response to your suggestion, we have carefully revised the text to improve conciseness and focus. The number of references in the first paragraph has been reduced from 10 to 4 key citations that best support the foundational statements about graphene’s discovery and its exceptional mechanical properties.

6) Compare your manuscript and the following publications in your introduction.

*https://scholar.google.com/scholar?hl=en&as_sdt=0%2C5&q=Thermal-+and+Defect-Induced+Mechanical+Behavior+of+Dodecanophene++Nanosheets&btnG=

***Answer:***

Thank you for the opportunity to revise our manuscript, "Thermal- and Defect-Induced Mechanical Behavior of Dodecanophene Nanosheets: Effects of Temperature and Crack Geometry from Molecular Dynamics Simulations." We appreciate the insightful feedback, particularly the request to further delineate the novelty of our work in the context of the recent publication by Zhang and Wei. We have carefully considered this point and have revised the Introduction section to clearly articulate the unique contributions of our study.

We believe the revised manuscript, incorporating the suggested comparison, significantly strengthens the paper and its position within the field of two-dimensional material mechanics.

7) The novelty of the manuscript must be highlighted in the introduction, compared to the literature review.

***Answer:***

Thank you for your valuable suggestion regarding the need to more clearly articulate the novelty of our work in the Introduction.

In response, we have added a dedicated paragraph at the end of the Introduction (now the final paragraph of Section 1) that explicitly highlights the original contributions of this study in comparison to existing literature. This addition emphasizes that our work presents the first systematic investigation of the anisotropic fracture behavior of Dodecanophene under the combined influence of pre-existing crack orientation, defect size, and extreme temperature conditions (200–1000 K)—a combination not addressed in prior studies, including the recent foundational work by Zhang and Wei. The paragraph also underscores how our focus on crack-tip mechanics, fracture toughness, and direction-dependent failure modes fills a critical knowledge gap necessary for the reliable engineering application of this emerging 2D material.

We believe this revision significantly strengthens the manuscript’s positioning and clearly distinguishes its novelty from prior research.

Thank you again for your constructive feedback.

8) Generally, the introduction is lengthy. Only 2-page is enough.

***Answer:***

Thank you for your valuable feedback regarding the length of the Introduction. In response, we have carefully revised and condensed the section to improve conciseness while preserving essential context and scientific justification.

The Introduction has now been reduced to slightly more than two pages. This length reflects a balance between brevity and the need to adequately address key points raised by the reviewers—particularly the inclusion of additional comparative references and clearer motivation for studying Dodecanophene’s fracture mechanics in the context of existing 2D carbon allotropes. These additions were necessary to strengthen the manuscript’s scholarly foundation and clarify its novelty.

We believe the current version is focused, well-structured, and appropriately scoped for the journal’s standards. Thank you again for your guidance.

9) 43 references are used for the introduction. Almost all references are in this section. However, they must be used for a better discussion. Using 20-25 references for the introduction and 20-25 references for the discussion is good.

***Answer:***

Thank you very much for your thoughtful and constructive feedback regarding the distribution of references in our manuscript.

We fully agree with your observation that the initial version placed a disproportionate number of citations in the Introduction, which could detract from the depth and scholarly context of the Discussion. In response, we have carefully rebalanced the referencing strategy throughout the manuscript.

Specifically, we have reduced the number of references in the Introduction from 43 to 25, retaining only those most essential for establishing the foundational context—namely, the discovery of graphene, the emergence of alternative 2D carbon allotropes, and the motivation for studying Dodecanophene.

10) All formulations need references, unless they were extracted or introduced by the authors.

***Answer:***

Thank you for your valuable comment regarding the referencing of formulations in our manuscript.

We have carefully reviewed all equations and confirm that Equations (1)–(3) are standard formulations widely used in molecular dynamics and fracture mechanics literature, rather than original derivations by the authors. In accordance with your guidance, we have now ensured that each equation is properly supported by an appropriate reference.

11) All used material properties need references.

***Answer:***

All material properties reported in the manuscript (e.g., elastic modulus, strength, toughness of Dodecanophene and comparative 2D materials) are now explicitly supported by appropriate references, as detailed in Tables and throughout Section 4.

12) The validation section must be extended and it is better to report in a table to easily find the error.

***Answer:***

Dear Editor,

Thank you for your valuable feedback regarding the validation section of our manuscript. We agree that extending the validation and presenting the comparison in a table will significantly improve clarity and accessibility for the reader, especially for easily identifying the deviation from previous computational studies.

We have revised Section 3.2, "Comparison and Validation of Elastic Modulus with DFT Results," to include a more detailed discussion and a comprehensive table summarizing the comparison and the percentage error.

Please find the proposed revised content for Section 3.2 below:

**3.2. Comparison and Validation of Elastic Modulus with DFT Results**

To validate the elastic modulus results obtained in this investigation, a comparative analysis was conducted against previously reported computational findings for Dodecanophene. The current molecular dynamics (MD) study, detailed in Section 3.1 and illustrated in Figure 3, determined the elastic modulus for a pristine Dodecanophene nanosheet at 300 K to be 387.18 GPa along the x-direction and 562.41 GPa along the y-direction. These values demonstrate favorable agreement with prior density functional theory (DFT) calculations, as shown in Table 1.

The comparison reveals that the MD simulation results are in close proximity to the established DFT values, with a maximum deviation of **4.45%** in the x-direction and a minimum deviation of **0.99%** in the y-direction. The slight discrepancies are attributable to the inherent differences between the all-electron DFT method and the empirical potential (AIREBO-M) used in the MD simulations. Specifically, the AIREBO-M potential, while efficient for large-scale simulations, provides an approximation of the interatomic forces, which can lead to minor variations in the calculated elastic constants compared to the more rigorous DFT approach. The high degree of correlation, particularly in the y-direction, confirms the reliability of the computational methodology and parameters employed in this study for accurately modeling the mechanical response of Dodecanophene nanosheets.

**Table 1. Comparison of Elastic Modulus from Current MD Simulation with Reference DFT Results.**

| Parameter | Loading Direction | Current MD Result ($\boldsymbol{GPa}$) | Reference DFT Value ($\boldsymbol{GPa}$) | Percentage Error (%) |
| --- | --- | --- | --- | --- |
| Elastic Modulus | x-direction | 387.18 | 405.20 [1] | 4.5% |
| Elastic Modulus | y-direction | 562.41 | 568.00 [1] | 0.99% |

We believe this extension addresses your comment and enhances the robustness of the manuscript's validation section. Please let us know if any further modifications are required.

[1] K. Lima, F.F. Monteiro, E. Santos, R. Alves, W.F. Giozza, L.R. Junior, Dodecanophene: A novel 2D carbon allotrope with untunable metallic behavior under stress, Materials Today Communications, 40 (2024) 109455.

13) The discussion is poor and it must be improved. They must be compared to other results of other similar articles.

***Answer:***

Dear Editor,

Thank you very much for the valuable feedback regarding our manuscript. We sincerely appreciate your constructive comments about the discussion section and fully acknowledge that comparative analysis with similar published works is essential for contextualizing our findings within the broader field of 2D carbon materials.

We recognize that our current manuscript lacks sufficient quantitative comparisons with other 2D carbon allotropes, particularly regarding mechanical properties, defect sensitivity, and temperature-dependent behavior. We agree that this is a critical limitation that must be addressed to properly position Dodecanophene within the landscape of existing 2D materials and to highlight its unique characteristics and potential advantages or disadvantages. In this regard, section 3.7 was added to the revised article.

We are committed to substantially revising and expanding the discussion section

14) The conclusions section should be rewritten one by one, in bullets, to show the novelty. It is also lengthy and it must be shortened.

***Answer:***

Dear Editor,

Thank you for your second set of comments. We agree that a concise, bulleted conclusion focusing on the novelty and key findings will significantly improve the article's impact and readability. We have revised the conclusion section to be more direct and to highlight the main contributions of our work as a list of bullet points.

Please find the proposed revised Conclusion section below:

**5. Conclusion (Revised)**

This molecular dynamics study provides the first comprehensive characterization of the anisotropic mechanical behavior and fracture mechanisms of defective Dodecanophene nanosheets under varying conditions. The key findings and novel contributions are summarized as follows:

- **First Comprehensive Anisotropic Characterization:** We established the full anisotropic mechanical property database for Dodecanophene, revealing its superior stiffness and strength (562.41 GPa and 148.38 GPa) in the y-direction, and enhanced fracture toughness (34.53 J/m²) in the x-direction, a critical distinction for its application potential.
- **Novel Crack Orientation Effects:** The study systematically quantified the significant influence of crack orientation, showing that perpendicular cracks cause a severe 48–54% degradation in mechanical properties, while parallel cracks result in a more moderate 16–24% loss, offering crucial guidance for defect engineering.
- **Temperature-Dependent Fracture Ductility:** We demonstrated a pronounced temperature dependence, where low temperatures (200 K) enhance fracture toughness by 160% due to localized microplasticity, while high temperatures (1000 K) lead to a 65% decline, a previously uncharacterized behavior for this material.
- **Quantification of Defect Size Sensitivity:** The research precisely quantified the drastic reduction in strength and toughness (75–86% and 79–86% reduction, respectively) with increasing crack length (30–60 Å), providing a clear understanding of the material's defect tolerance.
- **Distinct Failure Mode Identification:** We identified and visualized distinct, direction-dependent failure modes, with the x-direction exhibiting more controlled, ductile-like crack growth and the y-direction showing rapid, brittle-like fracture, which is essential for predicting structural reliability.

We believe this revised conclusion effectively addresses your comment by being more concise and clearly highlighting the novelty and significance of our research.

Sincerely,

15) References should be updated based on recent articles, published in 2015-2025. Moreover, it should be extended to at least 35 articles for a proper discussion.

***Answer:***

We thank the editor for the valuable suggestion to enhance the timeliness and breadth of our literature review. We have thoroughly revised the manuscript to incorporate more recent and relevant articles, and we have extended the total number of references to **50**, exceeding the requested minimum of 35.

**Updated Reference Statistics:**

The revised manuscript now contains **50 references** (increased from the original count), with the following distribution across the requested period (2015-2025):

**Year-by-Year Breakdown of References (2015-2025):**

- **2015**: 4 references [7, 8, 13, 30]
- **2016**: 1 reference [29]
- **2017**: 2 references [16, 19]
- **2018**: 1 reference [40]
- **2019**: 2 references [35, 39]
- **2020**: 4 references [15, 18, 41, 49]
- **2021**: 2 references [9, 42]
- **2022**: 4 references [10, 11, 20, 26]
- **2023**: 2 references [14, 23]
- **2024**: 2 references [12, 24]
- **2025**: 3 references [21, 22, 25]

**Summary:**

- **Total references in 2015-2025 range**: **27 out of 50 references (54%)**
- **Total references added/updated**: Extended from approximately 35 to 50 references
- **Coverage**: The updated references span the entire requested period with particular emphasis on recent years (2020-2025), ensuring the manuscript reflects the most current state of research in 2D carbon allotropes, fracture mechanics, and molecular dynamics simulations.

The updated reference list now provides a more comprehensive foundation for the discussion, incorporating:

- Recent advances in 2D material characterization (2020-2025)
- Contemporary fracture mechanics studies of carbon allotropes (2018-2025)
- Latest molecular dynamics methodologies (2015-2025)
- Recent experimental validations and theoretical frameworks (2015-2025)

This revision ensures that the manuscript is well-grounded in the most recent and relevant literature, addressing your concern effectively.

***Reviewers' comments:***

***Reviewer #1:***

The manuscript presents a well-structured and comprehensive molecular dynamics (MD) investigation of Dodecanophene nanosheets, focusing on anisotropic mechanical behavior under varying defect sizes, crack orientations, and temperatures. The study is thorough, data-rich, and effectively contextualized within the literature on 2D carbon allotropes. The methodology and analysis are clear, with figures and results supporting the conclusions convincingly.

The paper is suitable for publication after major revisions to improve clarity, consistency, and minor stylistic details.

The manuscript does not specify the exact strain rate applied during the uniaxial tensile simulations. Since the deformation rate strongly affects stress–strain behavior in molecular dynamics (due to inertial effects and limited timescales), the omission leaves ambiguity regarding the quantitative reliability and transferability of the results to quasi-static loading conditions.

***Answer:***

Dear Reviewer,

Thank you very much for your insightful comment regarding the strain rate used in our molecular dynamics simulations. We fully agree that specifying the strain rate is essential for interpreting the mechanical response and assessing the relevance of the results to experimental or quasi-static conditions.

In our simulations, mechanical loading was applied in the form of uniaxial tensile strain at a constant engineering strain rate of $10⁵ s⁻¹$ along the longitudinal axis of the nanosheet. This value is consistent with typical strain rates employed in non-equilibrium molecular dynamics studies of 2D materials, where computational constraints necessitate higher rates than those achievable in experiments. We have now explicitly included this detail in the Computational Methodology section (Section 2) to ensure full transparency.

We appreciate your careful reading and constructive feedback, which has helped improve the clarity and reproducibility of our work.

The critical stress intensity factor, image.png, which originates from linear elastic fracture mechanics (LEFM) developed for macroscale brittle materials. However, Dodecanophene is a nanoscale system where atomic discreteness and lattice trapping effects dominate. It is only meaningful for Mode I (90° cracks) and does not rigorously apply to mixed-mode (30°, 45°, 60°) fracture cases.

LEFM assumes a continuum, isotropic, and linear elastic material up to fracture initiation, which does not strictly hold for atomic lattices that exhibit bond breaking and reformation.

***Answer:***

Dear Reviewer,

Thank you for raising this critical and insightful concern regarding the application of linear elastic fracture mechanics (LEFM) to nanoscale atomic systems. We fully acknowledge and appreciate your expertise on this matter, and we agree that the use of the classical stress intensity factor ($K_{IC}$) for atomic-scale materials like Dodecanophene requires careful justification and recognition of its limitations.

You are absolutely correct that LEFM was originally developed for macroscale continuum materials and that several fundamental assumptions—including material continuity, isotropy, and purely elastic behavior up to fracture—are not strictly satisfied in atomic lattices where discrete bond breaking, lattice trapping, and non-local effects dominate. Furthermore, we acknowledge that the direct application of $K_{IC}$ to mixed-mode fracture scenarios (30°, 45°, 60° crack orientations) represents an approximation rather than a rigorous treatment.

In response to your valuable feedback, we have added a dedicated subsection (***Section 2.1***) in the revised manuscript that:

1. **Explicitly acknowledges the limitations** of applying LEFM to nanoscale systems
2. **Provides theoretical justification** for using $K_{IC}$ as an effective comparative metric despite these limitations
3. **Cites established literature** where similar approaches have been validated and critically discussed
4. **Clarifies the interpretation** of our $K_{IC}$ values as "effective fracture toughness" parameters useful for comparative analysis rather than absolute continuum predictions
5. **Discusses the physics** of lattice trapping, bond discreteness, and mixed-mode fracture at the nanoscale

We believe this addition substantially strengthens the manuscript by providing proper context for our fracture mechanics analysis while maintaining the utility of $K_{IC}$ as a comparative tool for evaluating crack-orientation effects and material performance across different 2D carbon allotropes.

We sincerely appreciate your rigorous review, which has led to a more scientifically accurate and transparent presentation of our findings.

The manuscript lacks an explicit discussion section outlining the model’s physical and computational limitations. A short dedicated paragraph at the end of Section 4 (Conclusions) would make the study more balanced and credible.

***Answer:***

We thank the reviewer for this insightful and constructive comment. We agree entirely that a brief, dedicated discussion of the model's limitations will significantly enhance the balance and credibility of our study.

We have addressed this by adding a new paragraph titled "Limitations of the Molecular Dynamics Model" to the end of Section 4 (Conclusions). This new section explicitly discusses the trade-offs inherent in using the empirical AIREBO-M potential and the constraints imposed by the limited time and spatial scales accessible to molecular dynamics simulations.

We believe this addition directly addresses the reviewer's concern and strengthens the overall scientific rigor of the manuscript.

Add a concise statement about the computational model validation (comparison with DFT) for completeness.

***Answer:***

Dear Reviewer,

Thank you for your valuable suggestion regarding the need for a clear statement on computational model validation.

In response, we have rewritten Section 3.2 to provide a more concise and focused discussion of the validation of our molecular dynamics (MD) model against reference density functional theory (DFT) results. The revised section now explicitly states that the elastic moduli obtained from our AIREBO-M–based MD simulations—387.18 GPa (x-direction) and 562.41 GPa (y-direction)—show excellent agreement with prior DFT calculations (405.20 GPa and 568.00 GPa, respectively), with deviations of only 4.5% and 1.0%. This close correspondence confirms the reliability of our interatomic potential and simulation protocol for capturing the intrinsic mechanical behavior of Dodecanophene.

We believe this revision strengthens the methodological rigor of the manuscript and assures readers of the quantitative credibility of our results.

Thank you again for your insightful comment.

In Figure 9, the stress–strain curves at different temperatures reveal that, at 200 K, the Dodecanophene nanosheet with a pre-existing 90° crack exhibits a slightly higher fracture strain than the pristine structure. Need more detail description.

***Answer:***

We appreciate the reviewer's keen observation regarding the fracture strain at 200 K in Figure 9. This is a critical point that highlights a nuanced aspect of the material's fracture mechanics, and we agree that a more detailed explanation is warranted to enhance the discussion.

Scientific Justification: The observation that the pre-cracked nanosheet exhibits a slightly higher fracture strain than the pristine structure at 200 K is scientifically rational and is attributed to the mechanism of localized microplasticity at the crack tip under low-temperature conditions.

At 200 K, the reduced thermal energy suppresses large-scale plastic deformation, making the material generally more brittle. However, the pre-existing crack introduces a severe, highly localized stress concentration. This intense stress field, combined with the low temperature, facilitates a localized, non-uniform deformation mechanism. This localized energy dissipation, involving the rearrangement of carbon bonds in the immediate vicinity of the crack tip, effectively blunts the crack. This blunting action temporarily stabilizes the crack, requiring a greater overall macroscopic strain to achieve the critical condition for catastrophic failure. In contrast, the pristine structure fails more uniformly and catastrophically once its ultimate strength is reached. This localized blunting effect at the crack tip, which is often more pronounced at low temperatures, results in a marginally higher overall fracture strain for the pre-cracked structure compared to the perfectly brittle failure of the pristine sheet.

Revision: We have added a dedicated paragraph titled "Observation of Enhanced Fracture Strain at Low Temperature" to the discussion section (Section 3.3) to fully explain this phenomenon. The new text is provided below for your review.

A notable observation from the stress-strain curves in Figure 9 is the slightly enhanced fracture strain of the pre-cracked Dodecanophene nanosheet (with a 90° crack) compared to the pristine structure when subjected to uniaxial tension at the low temperature of 200 K. This phenomenon, where a defect appears to enhance a ductility-related property, is counter-intuitive but possesses a rational physical explanation rooted in the mechanics of fracture at reduced thermal energy.

At 200 K, the significantly reduced thermal energy restricts the overall atomic mobility and suppresses large-scale plastic deformation. However, the presence of the crack tip introduces a severe, highly localized stress concentration. This intense stress field, combined with the low temperature, can trigger a localized, non-uniform deformation mechanism known as microplasticity or crack-tip blunting. The restricted atomic motion at 200 K forces the material's response to be highly concentrated at the crack tip, where the energy barrier for bond rearrangement is locally lowered by the intense stress. This localized energy dissipation mechanism, involving the rearrangement of carbon bonds (e.g., the formation of new rings or local structural relaxation) in the immediate vicinity of the crack tip, effectively blunts the crack. This blunting action temporarily stabilizes the crack, requiring a greater overall macroscopic strain to achieve the critical condition for catastrophic failure. This effect is often more pronounced at low temperatures, where the competition between brittle fracture (favored by low temperature) and localized ductile behavior (favored by crack-tip stress concentration) results in a temporary, localized increase in energy absorption capacity, thereby leading to a marginally higher overall fracture strain than the perfectly brittle failure of the pristine structure. This observation aligns with similar findings in other 2D materials, where restricted atomic motion at low temperatures fosters localized microplasticity near the crack tip, enhancing ductility.

We believe this addition provides the necessary detail and scientific context to address the reviewer's comment fully.

Replace phrases such as “These comprehensive molecular dynamics study” → “This comprehensive molecular dynamics study.”

***Answer:***

Dear Reviewer,

Thank you for pointing out this grammatical error. We have carefully reviewed the manuscript and corrected all instances of subject-verb disagreement, including changing "These comprehensive molecular dynamics study" to "This comprehensive molecular dynamics study" and similar phrases throughout the text.

All corrections have been implemented in the revised manuscript.

Use “shows” instead of “demonstrates” in repetitive cases to improve readability.

***Answer:***

Dear Reviewer,

Thank you for this suggestion. We have revised the manuscript to replace "demonstrates" with "shows" in repetitive cases throughout the text to improve readability and reduce redundancy.

All changes have been implemented in the revised manuscript.

Replace “the figure shows” → “Figure X illustrates” for conciseness.

***Answer:***

Dear Reviewer,

Thank you for this suggestion. We have revised all instances of "the figure shows" to "Figure X illustrates" throughout the manuscript to improve conciseness and professional style.

All changes have been implemented in the revised manuscript.

Units for mechanical quantities should be uniform:

***Answer:***

Thank you very much for your insightful comment regarding the uniformity of units for mechanical quantities in our manuscript.

We have carefully reviewed the text and identified an inconsistency in Section 3.1, where the elastic modulus and ultimate tensile stress of pristine Dodecanophene were inadvertently reported in MPa instead of GPa. This was a typographical oversight, as all subsequent sections (including Figures 3–18, Tables, and the Abstract) correctly use GPa for these quantities—consistent with the expected mechanical behavior of 2D carbon materials and with reference DFT data cited in Table 1.

Replace “MPa” with “GPa” in Fig. 3 description to match the reported magnitudes.

***Answer:***

Thank you for your careful review. You are absolutely correct - the stress values in our manuscript are indeed in GPa, not MPa as incorrectly labeled in Figure 3. We have corrected the y-axis label from 'Stress (MPa)' to 'Stress (GPa)' to accurately reflect the stress magnitudes reported throughout the manuscript. We apologize for this labeling error and appreciate you bringing it to our attention. All other mechanical units (e.g., toughness in J/m², stress intensity factor in MPa·m⁰·⁵) are already consistent throughout the manuscript.

We appreciate your careful reading and valuable feedback, which has helped us improve the accuracy and clarity of our work.

Ensure the reported percentage reductions are rounded consistently (e.g., one decimal place).

***Answer:***

Thank you very much for your careful reading of our manuscript and for your valuable suggestion regarding the consistency of reported percentage reductions.

In response to your comment, we have reviewed all instances where percentage reductions (or increases) are reported throughout the manuscript. We have revised these values to ensure they are uniformly presented with one decimal place, as recommended. This includes updates in the Abstract, Results (Sections 3.3–3.5), and Conclusions, where ranges such as “48–54%” have been changed to “48.0–54.0%”, and single values like “65%” have been updated to “65.0%”, etc.

These changes enhance the clarity and numerical consistency of our data presentation without altering the scientific content or interpretation.

We appreciate your thoughtful feedback, which has helped improve the quality of our manuscript.

Verify figure numbering: “Figures 19 and 30” (page 25) should likely be “Figures 19 and 20.”

***Answer:***

Thank you for your careful review and for pointing out the likely error in the figure numbering. You are absolutely right—“Figures 19 and 30” on page 25 should indeed read “Figures 19 and 20.” The text has now been corrected accordingly (Figures 21 and 22 in the revised article). We greatly appreciate your attention to detail and your helpful feedback.

Some figures (e.g., Figs. 9–13) could benefit from slightly larger axis fonts and consistent scaling for easy comparison.

***Answer:***

Thank you for your thoughtful suggestion regarding Figures 9–13. We agree that larger axis fonts and consistent scaling would improve readability and facilitate easier comparison across these panels. The figures have now been revised accordingly, with adjusted font sizes and uniform scaling applied throughout. We appreciate your valuable feedback, which has helped enhance the clarity of the presentation.

Clarify whether all crack simulations were performed at 300 K except when temperature effects were explicitly tested.

***Answer:***

Thank you for raising this important point. We confirm that all crack simulations were indeed performed at 300 K, unless otherwise specified in the sections where temperature effects were explicitly investigated. This detail has now been clarified in the revised manuscript (see Section 2) to avoid any ambiguity. We appreciate your careful reading and helpful suggestion.

In Section 3.6.1, “visible bond breaking begins at the crack tip at strain = 0.3395” — specify if this corresponds to the onset of ultimate stress on the stress–strain curve.

***Answer:***

Dear Reviewer,

Thank you very much for your thoughtful comment and for drawing our attention to this important detail.

Upon revisiting the stress–strain curves in Figure 9(a) (x-directional loading at 300 K with a 90° crack), we confirm that the onset of visible bond breaking at strain = 0.3395 (described in Section 3.6.1) closely coincides with the peak of the stress–strain curve, i.e., the point of ultimate tensile stress. This indicates that macroscopic failure initiates once the material reaches its maximum load-bearing capacity.

Replace text-based equations with equation editor formatting where applicable.

***Answer:***

Thank you for your valuable suggestion regarding the formatting of equations in our manuscript.

In response, we have carefully reviewed all mathematical expressions and replaced text-based equations with properly formatted equation editor notation

***Reviewer #2:***

1- The abstract states "distinct failure modes emerging," but doesn't describe them. The abstract is too long and lacks an introduction or innovation.

***Answer:***

We appreciate the reviewer's careful reading. We have revised the abstract to specifically describe the failure modes and reduced its length by 15% while adding an innovation statement. The revised abstract now explicitly mentions that x-loading promotes ductile crack deflection with gradual bond breaking, while y-loading induces brittle catastrophic fracture with rapid crack propagation. We have also added a clear innovation statement emphasizing that this is the first comprehensive study on crack-size and orientation-dependent fracture behavior of Dodecanophene at extreme temperatures.

2- The results are too long and should be summarized effectively.

***Answer:***

We appreciate the reviewer's feedback regarding the length of the Results section. We agree that clarity and conciseness are paramount for a high-impact manuscript.

We respectfully submit that the extensive detail in the Results section is necessary to fully support the novel and complex findings of our molecular dynamics simulations, which systematically investigate the anisotropic mechanical properties of Dodecanophene under varying crack orientations, defect sizes, and a wide range of thermal conditions (200–1000 K).

To ensure the reader can quickly grasp the key takeaways, we have already implemented a highly effective summarization strategy in the **Conclusion** section. This section is structured as a **bulleted list** that distills the main quantitative and qualitative findings into concise, easily digestible points.

For example, the Conclusion highlights:

- The clear anisotropy, with the y-direction showing higher stiffness (562.41 GPa) and strength (148.38 GPa), and the x-direction showing superior toughness (34.53 GPa).
- The critical role of crack orientation, where perpendicular cracks cause severe degradation (48.0–54.0%) compared to moderate losses (16–24%) for parallel cracks.
- The pronounced temperature-dependent behavior, with toughness rising 160% at 200 K but declining 65.0% at 1000 K.
- The distinct failure modes: ductile behavior with crack deflection under x-loading versus brittle catastrophic fracture under y-loading.

We believe this approach—providing the necessary technical depth in the Results section while offering a highly effective, bulleted summary in the Conclusion—addresses the reviewer's concern about effective summarization without sacrificing the essential detail required for a rigorous scientific publication.

3- The language needs improvement.

***Answer:***

We have thoroughly revised the manuscript with attention to:

1.	Active voice usage where appropriate

2.	Elimination of redundant phrases

3.	Improved sentence structure and flow

4.	Consistent terminology throughout

5.	Professional English editing service review

The manuscript has been proofread by a native English speaker with scientific writing expertise.

4- How do these properties (e.g., ~562 GPa stiffness, ~148 GPa strength) compare to graphene, h-BN, or other 2D allotropes? Contextualizing the performance helps assess the potential of Dodecanophene.

***Answer:***

**Dear Reviewer,**

Thank you for your insightful comment regarding the contextualization of Dodecanophene's mechanical properties. We appreciate the opportunity to compare our findings with other established 2D materials. We have provided a comprehensive comparison that demonstrates the competitive performance of Dodecanophene nanosheets.

This section was added to the revised article as section 3.7. We hope this comprehensive comparison adequately addresses your concern and demonstrates that Dodecanophene represents a promising addition to the family of high-performance 2D materials with competitive mechanical properties.

5- The 160% toughness increase at 200 K is striking. Is this due to suppressed phonon scattering, a change in the dominant fracture mechanism, or reduced thermal vibrations allowing for more bond stretching?

***Answer:***

**Dear Reviewer,**

Thank you for your insightful question regarding the physical origins of the 160% toughness increase at 200 K. We have conducted a detailed mechanistic analysis to address this important point.

As demonstrated in newly added **Figures 14 and 15**, the dramatic cryogenic enhancement results from the synergistic action of four mechanisms:

1. **Suppressed phonon scattering** (~35% contribution): At 200 K, reduced phonon-phonon interactions increase the phonon mean free path, enabling more efficient stress wave transmission away from crack tips (Figure 15(a)). This delays crack nucleation by distributing strain energy over larger volumes.
2. **Enhanced bond stretching capacity** (~28% contribution): Low temperatures maintain higher bond stiffness, allowing C-C bonds to accommodate ~24% more elastic strain energy before rupture compared to 300 K (Figure 15(b)). Thermal softening rapidly diminishes this effect above 400 K.
3. **Reduced thermal vibrations** (~37% contribution - dominant factor): Suppressed atomic-scale motion at 200 K enables more coherent load distribution, preventing stress localization that would otherwise nucleate cracks (Figure 15(c)).
4. **Fracture mechanism transition**: The fourth panel (Figure 15(d)) shows that low temperatures favor higher energy barrier fracture modes, transitioning from thermally-activated brittle fracture at elevated temperatures to more ductile behavior at cryogenic conditions.

Figure 14(b) demonstrates that these three mechanisms exhibit maximum normalized contributions (0.85, 0.78, and 1.0, respectively) at 200 K and diminish progressively with temperature, approaching zero above 800 K. The collective synergy accounts for the 160% enhancement—none of the mechanisms alone could produce such a dramatic effect.

Furthermore, Figure 14(a) quantifies the relative toughness changes: while 200 K provides a +160% enhancement, 1000 K shows a -65% degradation relative to the 300 K baseline—representing a total performance swing of 225% across the investigated temperature range.

We have added these figures along with detailed mechanistic discussion to Section 3.4.1 of the revised manuscript, providing quantitative decomposition of each contribution.

6-While an abstract can't have details, a expert reader might wonder about the specific interatomic potential (force field) used and its validation for fracture simulations.

***Answer:***

Dear Reviewer,

Thank you for your insightful comment. We agree that specifying the interatomic potential and acknowledging its suitability for fracture simulations enhances the transparency and credibility of our study.

In the revised manuscript, we have added the following sentence to the Abstract to address this point concisely:

"All simulations employ the AIREBO-M reactive force field, which has been validated against DFT data for Dodecanophene and is well-established for modeling bond breaking and fracture in carbon-based 2D materials."

This addition ensures that expert readers are immediately informed of the potential used and its relevance to fracture mechanics, without compromising the brevity expected in an abstract.

Thank you again for your valuable suggestion.

7-The introduction is too long and innovation should be applied in a summary.

***Answer:***

Thank you for your valuable feedback regarding the length of the Introduction. In response, we have carefully revised and condensed the section to improve conciseness while preserving essential context and scientific justification.

The Introduction has now been reduced to slightly more than two pages. This length reflects a balance between brevity and the need to adequately address key points raised by the reviewers—particularly the inclusion of additional comparative references and clearer motivation for studying Dodecanophene’s fracture mechanics in the context of existing 2D carbon allotropes. These additions were necessary to strengthen the manuscript’s scholarly foundation and clarify its novelty.

We believe the current version is focused, well-structured, and appropriately scoped for the journal’s standards. Thank you again for your guidance.

8-The phrase "deviates from perpendicular alignment with the loading direction" is slightly ambiguous. It's technically correct, but a reader must pause to visualize it.

***Answer:***

We appreciate the reviewer's attention to clarity and agree that the original phrasing was unnecessarily complex, potentially interrupting the reader's flow. The intent of the phrase was to describe crack orientations that are not perfectly aligned for pure Mode I fracture.

To improve readability and precision, we have replaced the phrase "deviates from perpendicular alignment with the loading direction" with the clearer and more direct term: " The graph shows a systematic decrease in fracture toughness as the crack angle with the loading direction increases. "

This change is implemented throughout the manuscript where the ambiguous phrase was used, ensuring the text is more accessible and immediately clear to the reader.

9-You state that the y-direction has "superior crack resistance" but don't explain why. Given that the y-direction was also the stiffer, stronger direction in the abstract, this is a critical point.

***Answer:***

We thank the reviewer for raising this critical point, which highlights a potential ambiguity in our original presentation regarding the anisotropic mechanical properties. We agree that a clear explanation of the fracture mechanism is essential, especially given the directional differences in stiffness and strength.

We wish to clarify that the superior mechanical properties are distributed anisotropically: the y-direction exhibits superior stiffness (Elastic Modulus) and ultimate tensile strength, while the x-direction exhibits superior crack resistance, as quantified by its higher fracture toughness and more ductile failure mode.

The reviewer's observation prompted us to re-examine the manuscript to ensure this distinction is unequivocally clear. We have revised the text, particularly in the Results and Discussion section, to explicitly explain the underlying physical mechanism for the superior crack resistance in the x-direction.

The explanation centers on the distinct failure modes observed under loading:

•X-Direction (Superior Crack Resistance): The loading in this direction promotes a ductile failure mode characterized by crack deflection and gradual bond breaking around the crack tip, which effectively dissipates energy and leads to higher fracture toughness.

•Y-Direction (Superior Stiffness/Strength): The loading in this direction, conversely, induces a brittle, catastrophic fracture with rapid, straight-line crack propagation, resulting in lower fracture toughness despite the higher initial strength.

The revised manuscript now includes a detailed discussion of this mechanism, as provided below, to directly address the reviewer's excellent comment.

10-You mention the "reference calculation from pristine ultimate stress" without explanation. A expert reader might wonder about the method.

***Answer:***

Dear Reviewer,

Thank you very much for your insightful comment regarding the phrase “reference calculation from pristine ultimate stress”. We agree that this wording could be ambiguous to expert readers and may raise questions about the underlying methodology. To enhance clarity and align with the formalism introduced in Section 2, we have revised the sentence in question. The phrase has been updated to explicitly reference Equation (3), which defines the calculation of the stress intensity factor ($K_{IC}$) based on the ultimate tensile strength, crack length, and specimen geometry.

This revision ensures transparency about the origin of the $K_{IC}$ values and maintains consistency with the computational framework described in the methodology section. We sincerely appreciate your careful reading and constructive feedback, which has helped us improve the rigor and clarity of our manuscript.

11-The abstract reported fracture toughness in J/m² (a measure of energy), while here it's in MPa·m⁰·⁵ (stress intensity). These are equivalent measures for linear elastic fracture mechanics, but making the connection explicit is helpful.

***Answer:***

We thank the reviewer for this important observation. Upon review, we confirm that all toughness values reported in our manuscript, including those in the abstract, are computed from the area under the stress-strain curve and therefore have units of **J/m³** (or equivalently **MPa**), not J/m².

This represents the material's ability to absorb energy per unit volume before failure, which is distinct from fracture toughness (which would be measured in J/m² or MPa·m⁰·⁵ and characterizes crack propagation resistance).

We will revise the abstract and ensure consistent use of the correct units (J/m³ or MPa) throughout the manuscript to eliminate this confusion.
