# Supplementary material for: Anisotropic mechanical properties of Dodecanophene nanosheets with pre-existing cracks by molecular dynamics simulation: Uncovering orientation- and temperature-induced variations
Source: PLoS One. 2026 Mar 6;21(3):e0339525. doi: 10.1371/journal.pone.0339525 (PMC12965698; doi:10.1371/journal.pone.0339525)
Supplement: Attachment — Submitted filename: Response2.docx [file pone.0339525.s003.docx]

PONE-D-25-51742R1

Anisotropic Mechanical Properties of Dodecanophene Nanosheets with Pre-Existing Cracks: Uncovering Orientation- and Temperature-Induced Variations

PLOS ONE

Still, the following issues must be addressed,

1) The keyword of "Molecular dynamics simulation" cannot be find in the title or the abstract. How can it be a keyword?

***Response:***

Thank you for this important observation. Molecular dynamics simulation was added to the title and abstract.

2) The whole abstract includes the results. Just the first sentence is the topic. The research method must be mentioned.

***Response:***

Thank you for this important observation. We have revised the abstract to include a clear description of the research methodology. The revised abstract now explicitly mentions the use of molecular dynamics simulations with the AIREBO-M reactive force field, along with the computational approach employed. Please see the revised abstract below.

3) What is "DFT" in the abstract? All abbreviations need to be defined at first mentioning.

***Response:***

We appreciate you pointing this out. We have now defined "DFT" as "Density Functional Theory" at its first mention in the abstract to ensure clarity for all readers.

4) What is the reference for a constant engineering strain rate of 100,000 /s?

***Response:***

Thank you for your comment regarding the constant engineering strain rate of 100,000 /s. We have now added the appropriate reference to support this value in the revised manuscript.

5) There is a section for 2.1 without any 2.2! How can it be?

***Response:***

Thank you for pointing this out. In the revised version of the manuscript, subsection 2.1 was intentionally removed to ensure a more streamlined and cohesive structure throughout the article. As a result, there is no longer a corresponding subsection 2.2, and the numbering has been adjusted accordingly for clarity and consistency.

6) Figure 3 is mentioned without any related text before it! Check all figures.

***Response:***

Thank you for identifying this issue. We have carefully reviewed the manuscript and ensured that all figures are properly introduced in the text before they are referenced. Specifically, we have added appropriate introductory text before the mention of Figure 3, and have verified that all other figures follow the same proper referencing structure throughout the manuscript.

7) The title of Figure 15 is so lengthy. The description of figures must be mentioned in the main text. Check all figure titles.

***Response:***

We agree that the caption for Figure 15 was excessively detailed. We have significantly shortened the caption while ensuring the essential information is retained. The detailed description has been moved to the main text where the figure is discussed. We have also reviewed all other figure captions to ensure they are concise and appropriate, with detailed descriptions provided in the main text.

8) The discussion is not just to describe the results. They must be compared to the other articles. Therefore, the discussion is still poor. This issue can be also understood from one reviewer's comment that Section 3.4.1 is added without any references.

***Response:***

Thank you for your valuable feedback. In response to the concern regarding the discussion section, we have significantly strengthened Section 3.7 by incorporating a comprehensive comparison of our findings with those reported in relevant recent literature. This addition addresses the need to contextualize our results within the broader scholarly conversation, rather than merely describing them.

Furthermore, this revision also responds to the reviewer’s comment about Section 3.4.1 lacking references; appropriate citations have now been integrated throughout the discussion to support our interpretations and comparisons.

9) I cannot see any highlighted references in the revised manuscript. No changes in references?

***Response:***

The newly added references in the revised version were indeed highlighted to facilitate identification, as per the journal’s guidelines for revisions.
